# Anticoagulant drugs with or without proton pump inhibitor and colorectal cancer risk: a population-based, case–control study

**DOI:** 10.1186/s12876-022-02314-w

**Published:** 2022-05-09

**Authors:** Pei-Huan Ho, Hung-Chun Hsiao, Chun-Wei Chen, Hui-Ming Chen, Siew-Na Lim, Chau-Ting Yeh, Chia-Jung Kuo, Wey-Ran Lin

**Affiliations:** 1 Department of Internal Medicine, Linkou Chang Gung Memorial Hospital, Taoyuan, Taiwan; 2 Liver Research Center, Linkou Chang Gung Memorial Hospital, Taoyuan, Taiwan; 3 Department of Gastroenterology and Hepatology, Linkou Chang Gung Memorial Hospital, 5, Fu-Shin Street, Taoyuan, 333 Taiwan; 4 Center for Big Data Analytics and Statistics, Linkou Chang Gung Memorial Hospital, Taoyuan, Taiwan; 5 Department of Neurology, Linkou Chang Gung Memorial Hospital, Taoyuan, Taiwan; 6 Chang Gung University College of Medicine, Taoyuan, Taiwan

**Keywords:** Colorectal cancer, Low-dose aspirin, Clopidogrel, Direct oral anticoagulant, Proton pump inhibitor

## Abstract

**Background:**

Low-dose aspirin and clopidogrel have demonstrated potential chemoprevention for colorectal cancer (CRC). Proton-pump inhibitors (PPI) are commonly prescribed with anticoagulation drugs, but the relationship between PPI and CRC is unclear. Moreover, evidence of CRC risk under direct oral anticoagulant (DOAC) is limited. This study aimed to investigate the effects of anticoagulation drugs combined with or without PPI on the risks of CRC in Taiwan.

**Methods:**

A retrospective case–control study of 1,024,227 cases based on the Chang Gung Research Database from 2010 to 2017 was performed. Clinical characteristics, indications, duration of anticoagulation and PPI use, and CRC occurrence data were collected. Logistic regression was employed to adjust for known confounders of CRC risk.

**Results:**

Monotherapy of clopidogrel decreased the risk of CRC (AOR 0.70; 95% CI 0.60–0.83), while no protective effect was observed in aspirin alone or aspirin plus clopidogrel. DOAC did not affect CRC significantly. The risk of CRC increased in patients with PPI (AOR 1.38; 95% CI 1.28–1.49) and PPI plus DOAC (OR 3.91; 95% CI 1.49–10.27), while PPI plus aspirin decreased the risk of CRC (OR 0.48; 95% CI 0.32–0.73). PPI plus clopidogrel showed no significant effect on the CRC.

**Conclusion:**

This study suggests clopidogrel alone and PPI plus aspirin offer a preventative benefit against CRC in the Taiwanese population studied. The same effect was not observed in DOAC. Moreover, a significant increase in CRC was observed in patients on PPI monotherapy and PPI plus DOAC, suggesting a possible risk.

**Supplementary Information:**

The online version contains supplementary material available at 10.1186/s12876-022-02314-w.

## Background

Colorectal cancer (CRC) is one of the most common types of cancer worldwide, with rising incidence rates reported in countries with high human development index [1]. In Taiwan, CRC ranks second in terms of incidence and third in terms of mortality for all types of cancers. In 2018, the incidence rate of CRC was 47.87/100,000 among females and 53.73/100,000 among males. As a leading cause of mortality, CRC is a growing burden on the healthcare system. Thus, it is important to understand risk factors that increase the chance of developing CRC.

Current evidence suggests low-dose aspirin reduces the risk of CRC, possibly via the inhibition of cyclo-oxygenase or platelet function [2]. Clopidogrel is another common antiplatelet agent, and several studies have also reported a protective effect from low-dose aspirin with or without clopidogrel [3, 4].

Due to the risk of gastrointestinal bleeding while using anti-platelet drugs, current guidelines recommend prevention with proton-pump inhibitor (PPI) treatment [5, 6]. Prescription of PPI has become more common in recent years. However, long-term safety of using PPI was still controversial, especially the risk of malignancy. PPI irreversibly blocks the secretion of gastric acid [7], which also results in pathological high level of gastrin [8]. The main concern of the carcinogenesis was related to systemic hypergastrinemia, which may promote the proliferation of epithelial cell [9]. However, the clinical relationship between CRC and PPI was yet to be determined [10–13]. Furthermore, there was a lack of literature of CRC incidence and combination of PPI plus anti-platelet agent use.

As another type of anti-thrombotic agent, direct oral anti-coagulant (DOAC) influences the coagulation cascade through inhibiting factor Xa or thrombin. PPI is as well prescribed with DOAC for preventing gastrointestinal bleeding. However, there was few evidences of DOAC with or without PPI affecting the risk of CRC [14].

While most studies related to aspirin or clopidogrel were designed for a non-Asian population, this study was performed from Taiwan population data. We also aimed to find out whether DOAC possesses the same protective effect as low-dose aspirin. Due to the increased prescription of PPI in recent years, clarifying the risk of CRC under PPI treatment has become a crucial issue. The combination PPI and anti-thrombotic therapies listed above were considered to reveal the relationship between drug usage and CRC incidence.

## Methods

### Study design

The Chang Gung Research Database (CGRD) was employed for this study. CGRD was a de-identified database derived from Chang Gung Memorial Hospital, which included seven medical institutes in Taiwan. Previous study observed CGRD had higher severity of comorbidities and prevalence of specific diseases [15]. This database contained case data including diagnoses, examination reports, and medication information.

This case control study was performed from January 1, 2010, to December 31, 2017. Patients who underwent either colonoscopy or abdominal CT scan were enrolled. Examination codes are listed in Additional file 1: Table S1. Exclusion criteria include CRC diagnosis one year before or after the study (n = 9714), unclear gender (n = 2), missing data of BMI (n = 298,444), and age younger than 20 years old (n = 66,894).

1,024,227 cases were included in the study and were grouped into CRC and non-CRC groups for retrospective analysis. The relationship between incidence of CRC with low-dose aspirin, clopidogrel, PPI, and direct oral anticoagulant (DOAC) therapy was analyzed. Low-dose aspirin was defined as a daily dosage of 75–100 mg. Ethics approval for this study was obtained from the Chang Gung Medical Foundation Institutional Review Board (IRB number: 201900776B0C502).

### Index date

Index date in the CRC group was the date of diagnosis, while the index date in the non-CRC group was the date of first examination. In both groups, drug usage was traced back to one year before the index date.

### Propensity score matching

We matched gender and age first, then year of index date between the two groups given the differing definitions for index date. The CRC group (n = 10,481) and non-CRC group (n = 41,924) were selected randomly at a 1:4 proportion.

### Potential confounding variables

Confounding variables were obtained one year before the index date, including lifestyle habits (smoking and alcohol consumption) and comorbidities. Comorbidities included hypertension, diabetes mellitus, cardiovascular disease, stroke, inflammatory bowel disease, GI bleeding. The ICD codes for these conditions are listed in Additional file 1: Table S2.

### Exposure definition

Exposure period was calculated and added individually from first prescription to drug suspension lasting more than 1 month (not calculating the days suspended). A new accumulation would be started at the next prescription. In each analytic group, the exposure periods of each drug were analyzed as follows.

When more than one drug was used for 3 months concurrently, each drug period over 3 months was summed individually. By comparing cumulative durations, the two most commonly-used drugs were selected as combination therapy. Residual cases were grouped into the monotherapy according to their most frequently used drug. Cases that did not use the drugs in the analytic group were classified as non-drug users.

### Statistical analysis

Drugs associated with risk of CRC were analyzed by conditional logistic regression to adjust for known confounders of CRC, including lifestyle habits and comorbidities. Crude and adjusted odds ratios were computed to show relationships between medication usage and risk of CRC along with a 95% CI. All analyses were performed under SAS software version 9.4 (SAS Institute), and the level of significance was set as *p* < 0.05.

## Results

### Baseline characteristics

10,481 CRC cases and 41,924 non-CRC cases were analyzed, with the characteristics of these cases listed in Table 1. Patients between 51 and 80 years of age accounted for 75.54% of all cases studied. The mean BMI level of the CRC and non-CRC group were 24.34 and 24.62. Confounding variables such as lifestyle habits and comorbidities were also considered. The most common comorbidity among the CRC group was hypertension, which accounted for 32.02% of cases. Diabetes mellitus was second, present in 18.98% of all CRC cases studied.

**Table 1:** Baseline case characteristics

Variables	Total (N = 52,405)	CRC (N = 10,481)	Non-CRC^a^ (N = 41,924)	*p* Value
Gender, n				1.0000
Male	30,760(58.70)	6152(58.70)	24,608(58.70)	
Female	21,645(41.30)	4329(41.30)	17,316(41.30)	
Age, mean (± SD), yr	65.18 ± 12.62	65.18 ± 12.62	65.18 ± 12.62	
Age group (n, %)				0.9997
< 51	5990 (11.43)	1196 (11.41)	4794 (11.86)	
51–80	39,585 (75.54)	7922 (75.58)	31,663 (75.52)	
> 81	6830 (13.03)	1363 (13.01)	5467 (13.04)	
BMI, mean (± SD), kg/m^2^	24.57 ± 3.88	24.34 ± 3.98	24.62 ± 3.86	
BMI class (n, %)				< 0.0001
< 24	24,187(46.15)	5113(48.78)	19,074(45.50)	
25–30	23,639(45.11)	4479(42.73)	19,160(45.70)	
> 30	4579(8.74)	889(8.48)	3690(8.80)	
Smoking (n, %)				0.4562
Past smoker	262(0.50)	215(0.51)	47(0.45)	
Current smoker	65(0.12)	55(0.13)	10(0.10)	
Nonsmoker	52,078(99.38)	41,654(99.36)	10,424(99.46)	
Alcohol (n, %)				0.1143
Alcoholism	570(1.09)	441(1.05)	129(1.23)	
Non-alcoholism	51,835(98.91)	41,483(98.95)	10,352(98.77)	
Comorbidities (n, %)				
Hypertension	13,985(26.69)	3356(32.02)	10,629(25.35)	< 0.0001
Diabetes Mellitus	8022(15.31)	1989(18.98)	6033(14.39)	< 0.0001
Cardiovascular disease	2106(4.02)	455(4.34)	1651(3.94)	0.0602
Stroke	1786(3.41)	396(3.78)	1390(3.32)	0.0195
Inflammatory bowel disease	61(0.12)	22(0.21)	39(0.09)	0.0017
GI bleeding	2905(5.54)	1405(13.41)	1500(3.58)	< 0.0001

^a^ Model was adjusted only for matching variables (age, gender, and year of index date), two groups classified present at 1:4 ratio

### Clopidogrel decreased the risk of CRC

Antiplatelet agents, including aspirin and clopidogrel, were analyzed in Table 2 under multivariate analysis. Monotherapy of the clopidogrel decreased the risk of CRC by 30% (AOR 0.70; 95% CI 0.60–0.83, *p* < 0.001). Aspirin plus clopidogrel or aspirin alone showed no significant effect on the risk of CRC.

**Table 2:** Relationship between low-dose aspirin, clopidogrel, PPI, and incidence of CRC

Variable	CRC (N = 10,481)	Non-CRC ^a^ (N = 41,924)	Crude OR (95%CI)	Adjusted OR^b^ (95%CI)
PPI	1195(11.40)	2824(6.74)	1.817(1.691–1.953)***	1.378(1.276–1.487)***
PPI + Low-dose aspirin	27(0.26)	120(0.29)	0.972(0.640–1.477)	0.564(0.364–0.872)**
PPI + Clopidogrel	38(0.36)	75(0.18)	2.192(1.482–3.241)***	1.162(0.765–1.764)
Low-dose aspirin	1040(9.92)	3922(9.36)	1.144(1.063–1.232)***	0.923(0.847–1.006)
Clopidogrel	217(2.07)	868(2.07)	1.080(0.928–1.257)	0.704(0.597–0.831)***
Low-dose aspirin + Clopidogrel	38(0.36)	154(0.37)	1.065(0.746–1.521)	0.726(0.499–1.058)
Non-drug user	7926(75.62)	33,961(81.01)	Ref	Ref

^a^Model was adjusted for matching variables (age, gender, and year of index date), two groups present at 1:4 ratio

^b^Model was adjusted for the following variables: age, gender, and year of index date; comorbidities and risk factors, including smoking, alcohol usage, hypertension, diabetes mellitus, cardiovascular disease, stroke, inflammatory bowel disease, GI bleeding

*p < 0.05, **p < 0.01, *** *p* < 0.001

### DOAC showed no protective effect against CRC

Four DOACs—apixaban, dabigatran, edoxaban and rivaroxaban—were considered for analysis (Table 3). The results showed that these DOACs were not associated with risk of CRC upon multivariate analysis.

**Table 3:** Relationship between DOAC and incidence of CRC

Variables	CRC (N = 10,481)	Non-CRC^a^ (N = 41,924)	Crude OR (95%CI)	Adjusted OR^b^ (95%CI)
*DOAC*				
Apixaban	7(0.07)	24(0.06)	1.173(0.505–2.722)	0.746(0.309–1.800)
Dabigatran	29(0.28)	77(0.18)	1.514(0.987–2.322)	1.250(0.802–1.948)
Edoxaban	4(0.04)	8(0.02)	2.007(0.604–6.668)	2.056(0.616–6.868)
Rivaroxaban	57(0.54)	146(0.35)	1.570(1.154–2.135)**	1.261(0.916–1.736)
Non-drug user	10,384(99.07)	41,669(99.39)	ref	ref

^a^Model was adjusted only for matching variables (age, gender, and year of index date), two groups present at 1:4 ratio

^b^Model was adjusted for the following variables: age, gender, and year of index date; comorbidities and risk factors, including smoking, alcohol usage, hypertension, diabetes mellitus, cardiovascular disease, stroke, inflammatory bowel disease, GI bleeding

*p < 0.05, **p < 0.01, ***p < 0.001

### PPI alone was associated with high risk of CRC

Table 2 reveals that the percentage of patients with PPI alone was higher in the CRC group compared to the non-CRC group (11.40% vs. 6.74%). Further multivariate analysis revealed that PPI monotherapy increased the risk of CRC by 38% (AOR 1.38; 95% CI 1.28–1.49, *p* < 0.001).

### PPI plus DOAC increased the risk of CRC, but PPI plus aspirin decrease the risk

This study also considered three combination therapies: PPI plus low-dose aspirin, PPI plus clopidogrel, and PPI plus DOAC (Table 4). The risk of CRC increased under a regimen of PPI plus DOAC (OR 3.91; 95% CI 1.49–10.27, *p* = 0.006), while PPI plus aspirin decreased the risk (OR 0.48; 95% CI 0.32–0.73, *p* = 0.005). PPI plus clopidogrel showed non-significant result on the CRC.

**Table 4:** Relationship between PPI combination therapy and CRC risk

Variable	Univariate logistic regression model			Multivariate logistic regression model^a^		
	OR	95%CI	*p* Value	OR	95%CI	*p* Value
PPI + Low-dose aspirin	0.799	0.540–1.184	0.2635	0.483	0.321–0.728	0.0005***
PPI + Clopidogrel	1.800	1.279–2.532	0.0007***	1.009	0.701–1.453	0.9607
PPI + DOAC	5.002	1.974–12.676	0.0007***	3.906	1.485–10.273	0.0058**

^a^Model was adjusted for the following variables: age, gender, and year of index date; comorbidities and risk factors, including smoking, alcohol usage, hypertension, diabetes mellitus, cardiovascular disease, stroke, inflammatory bowel disease, GI bleeding

*p < 0.05, **p < 0.01, ***p < 0.001

## Discussion

To our knowledge, this is the first study on the risk of CRC due to low-dose aspirin, clopidogrel, and DOAC therapy with PPI in combination. In this study, clopidogrel alone (AOR 0.70; 95% CI 0.60–0.83) and PPI plus aspirin (OR 0.48; 95% CI 0.32–0.73) reduced the risk of CRC, while PPI monotherapy (AOR 1.38; 95% CI 1.28–1.49) and PPI plus DOAC combination therapy (OR 3.91; 95% CI 1.49–10.27) increased the risk of CRC. There was no significant difference in risk of CRC among other monotherapies or drug combinations studied.

As a non-selective cyclo-oxygenase inhibitor, low-dose aspirin has shown to provide a protective effect against CRC in multiple studies [2, 16–21]. Indeed, the US Preventive Services Task Force recommended daily low-dose aspirin as a chemo-preventive agent against CRC in 2017 [16]. Modulation of aspirin cyclo-oxygenase activity is considered to be the main mechanism by which CRC risk is reduced. However, recent evidence also suggests that platelets promoted tumorigenesis and metastasis. Accordingly, the antiplatelet effect of aspirin is also suspected as a protective mechanism [22, 23]. In our study, monotherapy with aspirin did not reduce the risk of CRC (AOR 0.92; 95% CI 0.85–1.01). Our study design was carefully reviewed and compared with other studies, and below two reasons may lead to the non-significant association between aspirin and the risk of CRC. Firstly, the exposure of aspirin in our study was only observed for one year. An updated meta-analysis in 2019 reported that the risk of CRC declined as the prolonged usage of aspirin [19]. The short-term follow up of drug exposure may lead to such result. Secondly, some aspirin users were grouped into the aspirin plus clopidogrel group in this study. As such, the monotherapy group may not be representative of all cases. Different categorization criteria invariably lead to different outcome.

Clopidogrel, as a prodrug, requires two-step metabolism to irreversibly inhibit the P2Y12 receptor. Few studies on the risk of CRC under clopidogrel therapy have been conducted. One nested case–control study performed in Spain from 2001 to 2014 reported a decreased risk of CRC under therapy clopidogrel alone or in combination with aspirin [3]. Another retrospective study specific to cases of type 2 diabetes mellitus was carried in Taiwan, reporting a decreased risk of CRC among patients taking clopidogrel with or without aspirin [4]. The cases enrolled in our study may be regarded as representative of the global Asian population at large, including with respect to comorbidities. Present study reported decreased risk of CRC by 30% under clopidogrel monotherapy, though no protective effect was noted under aspirin plus clopidogrel.

Evidence on the link between DOAC and colorectal cancer is limited. One case–control study performed in the United Kingdom from 2011 to 2017 considered cases of non-valvular atrial fibrillation. In this study, 28,800 cases were followed, finding 1119 instances of cancer. Usage of DOAC did not increase the overall risk of malignancy, though a detrimental association between CRC and DOAC was observed upon subgroup analysis [14]. In our study, DOAC did not significantly affect the risk of CRC. This is the first study based on an Asian population and not limited to cases of atrial fibrillation. Due to the limited evidence currently available, further studies are required to construct a clearer relationship between DOAC and the risk of CRC.

PPI inhibits the H+/K+ adenosine triphosphatase pump to block the secretion of gastric acid, which leads to pathological elevation of gastrin [7, 8]. Several studies reported that systemic hypergastrinemia may promote the proliferation of epithelium, resulted in the carcinogenesis effect [9, 24, 25]. Recently, an association between imbalance of the gut microbiota and CRC was reported [26]. Alternation of gut microbiota was observed in the users of PPI, which was suspected to be another reason of tumorigenesis [27]. However with PPI alone, the epidemiology researches about the relationship to CRC risk remain controversial. Two researches, including a nested case control study at the United States in 2020 and a United Kingdom study in 2021, reported that PPI did not increase the risk of CRC [12, 13]. Conversely, a Korean study in 2017 reported an increased risk of CRC among patients on PPI therapy [10]. In our study, PPI monotherapy increased the risk of CRC (AOR 1.38; 95% CI 1.28–1.49). Our finding is consistent with the Korean population-based study from 2017. Both study design based on Asian population may lead to the similar result. The possible carcinogenesis effect of PPI may relate to hypergastrinemia or changing in gut microbiota. Further evidences were required for the mechanism. Evidence on combination PPI therapy is limited. The present study found that PPI plus DOAC (OR 3.91; 95% CI 1.49–10.27) increased the risk of CRC, whereas PPI plus aspirin decrease the risk of CRC (OR 0.483; 95% CI 0.32–0.73). PPI plus aspirin did not significantly affect the risk of CRC.

There are several merits to our study. The cases under consideration were based on the Taiwan population, making this is the first Asian population-based study on CRC risk due to DOAC treatment. Owing to different regional lifestyles, results obtained from a given region may require further interpretation. Secondly, our study did not limit case enrollment to particular disease. The inclusion of general cases in studying CRC risk under DOAC or clopidogrel plus low-dose aspirin therapy is novel. Cases in our study were also matched with confounding variables so that results may be applied to the general population. Finally, there is no research currently available on PPI combination therapy. Due to the rising rate of PPI prescription, the associated risk of CRC has become an issue of focus.

Limitations to our study are herein listed. First, different drug dosages and durations were not considered. Second, medication adherence was not considered due to the limitation of database. Third, our study was retrospective and case-controlled. The methodological quality of retrospective, case–control studies is inferior to that of randomized control and/or prospective studies. Granted, it is impractical to follow-up with cancer cases through a prospective study design.

## Conclusion

In this study based on East-Asian population, clopidogrel alone and PPI plus aspirin were found to decrease the risk of CRC. Conversely, PPI alone and PPI plus DOAC increased the risk of CRC. No significant difference was reported with respect to CRC risk and other therapeutic drugs.

## Supplementary Information

**Additional file 1.** **Table S1.** Definition of Codes for theChang Gung Memorial Hospital Examination Code Systems. **Table S2.** Definition of Codes for Different Coding System.

## Data Availability

The datasets used and/or analyzed during the current study are available from the corresponding author on reasonable request.

## Abbreviations

CRC: Colorectal cancer
PPI: Proton pump inhibitor
DOAC: Direct oral anticoagulant
CGRD: Chang Gung Research Database
