# Supplementary material for: Anticoagulant drugs with or without proton pump inhibitor and colorectal cancer risk: a population-based, case–control study
Source: BMC Gastroenterol. 2022 May 9;22:225. doi: 10.1186/s12876-022-02314-w (PMC9082832; doi:10.1186/s12876-022-02314-w)
Supplement: Supplementary file 1 — Additional file 1. Table S1. Definition of Codes for theChang Gung Memorial Hospital Examination Code Systems. Table S2. Definition of Codes for Different Coding System. [file 12876_2022_2314_MOESM1_ESM.docx]

**Appendix**

| Supplementary Table 1. Definition of Codes for the Chang Gung Memorial Hospital Examination Code Systems | |
| --- | --- |
| Examination | Examination Code |
| Polypectomy(LGI) | M23-006/ M23-013a |
| Colonofiberscopy | M23-013 |

| Supplementary Table 2. Definition of Codes for Different Coding Systems | | |
| --- | --- | --- |
| Comorbidities | ICD-9-CM | ICD-10 |
| Hypertension | 401-405 | I10, I11, I12, I13, I15 |
| Diabetes Mellitus | 250.0-250.9 | E10-E11 |
| Cardiovascular disease | 443, 410, 413, 272 | I73, I21, I20, E78 |
| Stroke | 434 | I63 |
| Inflammatory bowel disease | 555-556 | K50-K51 |
| GI bleeding | 530.21, 530.7, 530.82, 531.0, 531.2, 531.4, 531.6, 532.0, 532.2, 532.4, 532.6, 533.0, 533.2, 533.4, 533.6, 534.0, 534.2, 534.4, 534.6, 535.01, 535.11, 535.21, 535.31, 535.1, 535.51, 535.61, 535.71, 537.83, 537.84, 578  562.02, 562.03, 562.12, 562.13, 569.3, 569.85, 569.86, 578 | I85.01, I85.11, K20.81, K20.91, K21.01, K22.11, K21.01, K25.0, K25.2, K25.4, K25.6,  K26.0, K26.2, K26.4, K26.6, K27.0, K27.2, K27.4, K27.6, K28.0, K28.2, K28.4, K28.6, K29.01, K29.21, K29.31, K29.41, K29.51, K29.61, K29.71, K29.81, K29.91  K50.011, K50.111, K50.811, K50.911, K51.011, K51.211, K51.311, K51.411, K51.511, K51.811, K51.911, K55.21, K57.01, K57.11, K57.13, K57.21, K57.31, K57.33, K57.41, K57.51, K57.53, K57.81, K57.91, K57.93, K62.5, K92.2 |
| Colorectal Cancer | 153, 154 | C18-C21 |
